# Association of Vitamin D With Periodontal Disease: A Narrative Review

**DOI:** 10.3290/j.ohpd.a44323

**Published:** 2020-04-01

**Authors:** Panagiotis Dragonas, Iosif el-Sioufi, Yiorgos A. Bobetsis, Phoebus N. Madianos

**Affiliations:** a Assistant Professor, Department of Periodontics, School of Dentistry, Louisiana State University Health Sciences Center, New Orleans, USA. Idea, performed literature review, wrote and proofread manuscript.; b Resident, Department of Periodontics, School of Dentistry, National and Kapodistrian University of Athens, Greece. Idea, performed literature review, wrote and proofread manuscript.; c Assistant Professor, Department of Periodontics, School of Dentistry, National and Kapodistrian University of Athens, Greece. Proofread manuscript, contributed to discussion and literature review.; d Professor, Department of Periodontics, School of Dentistry, National and Kapodistrian University of Athens, Greece. Proofread manuscript, contributed to discussion and literature review.

**Keywords:** periodontal disease, vitamin D concentration, vitamin D supplementation, vitamin D receptor, polymorphism, periodontal inflammation

## Abstract

**Purpose:**

To present a review of available literature on the association of vitamin D and periodontal disease.

**Materials and Methods:**

A thorough search of articles was carried out on the databases PUBMED and MEDLINE regarding vitamin D and periodontal disease. The selected literature included cross-sectional, case-control and prospective and retrospective cohort studies. The main aspects of the association evaluated were a) the association of 25(OH)D and 1,25(OH)2D3 with periodontal disease severity, periodontal disease progression and tooth loss, b) the effect of vitamin D supplementation on periodontal health and c) the association of vitamin D receptor polymorphisms with periodontal disease. A brief overview of the biological mechanisms linking periodontal disease with vitamin D was also included.

**Results and Conclusions:**

There is conflicting evidence regarding the effects of 25(OH)D on periodontal disease severity, progression and tooth loss, with some studies reporting beneficial effects of higher 25(OH)D serum concentrations on periodontal health and tooth retention, whereas others could not find such an association. Limited evidence also supports a positive association between 1,25(OH)2D3 and periodontal health as well as a trend towards better periodontal health with vitamin D supplementation. Finally, various vitamin D polymorphisms were associated with chronic and aggressive periodontitis, with different outcomes reported for the various ethnic populations assessed.

Periodontitis is an infectious disease that leads to the destruction of the supporting tissues of the teeth. Today, it is widely accepted that connective tissue destruction and alveolar bone resorption are mainly host mediated through the release of pro-inflammatory cytokines and inflammatory mediators by local tissues and immune cells as a reaction to the bacterial challenge.^52^ Hence, environmental and genetic factors that modify the host’s immune response against periodontal pathogens may affect the progression and severity of periodontal disease.^34,35^ Several studies published over the past few years support the notion that vitamin D may constitute one such factor. Vitamin D is mainly produced by human skin after its exposure to ultraviolet radiation through sunlight, and it is also supplied through the individual’s diet (vitamin D2 and D3). The main form of vitamin D in plasma is 25-hydroxyvitamin D (25(OH)D) and represents the dominant measure of the body’s vitamin D storage, but has more limited biological functions compared to its active metabolite, 1,25-hydroxyvitamin D (1,25(OH)2D3).^4^ Vitamin D plays a significant role in a variety of physiological processes, including bone and calcium metabolism, immune functions and cellular growth and differentiation. Specifically, its major function is the maintenance of serum calcium and phosphorus concentrations within a normal range.^25^ When serum calcium levels drop below normal, the synthesis of vitamin D is increased, resulting in an increase in the absorption of calcium from the intestine and an increase in the osteoclastic activity in the bone in order to release stored calcium to the circulation. There is evidence that vitamin D also has anti-inflammatory and anti-microbial effects as it modulates the production of cytokines by immune cells and the secretion of anti-bacterial peptides by cells of the monocyte-macrophage lineage.^24,63^ As several studies have reported an association between osteoporosis or low bone density and alveolar bone and tooth loss,^12,51^ vitamin D, through both its effects in bone metabolism/bone mineral density (BMD) and the immune system, could influence the development of periodontal disease.^37^ Due to this potential association, vitamin D receptor gene polymorphisms have also been studied as potential genetic risk factors for periodontal disease in different ethnic populations with conflicting results so far.^17,53,56^ The aim of this review is to present the available information concerning all aspects of the possible association between periodontal disease and vitamin D and/or its receptor.

## Potential Mechanisms Linking Vitamin D and Periodontal Disease

**Fig 1:**
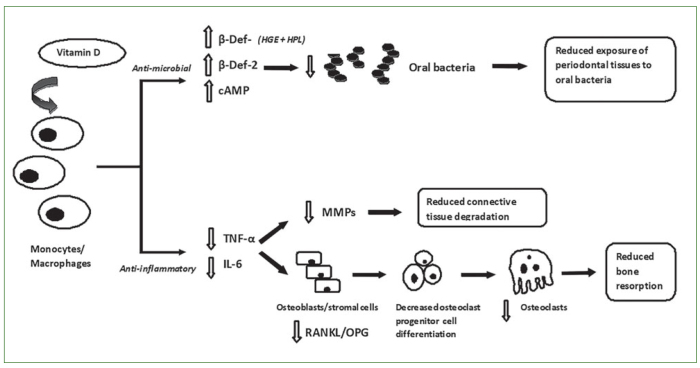
Possible protective mechanisms of vitamin D against periodontal disease. Vitamin D may protect against periodontal disease by activating two biological pathways: A) The anti-microbial pathway: The anti-microbial effect of vitamin D is the result of the binding of 1,25(OH)2D3 with the VDR/VDRE complex which induces the production of cAMP and β-Def-2 and β-Def-3 by macrophages, monocytes, human gingival epithelium (HGE) cells and human periodontal ligament (HPL) cells. These anti-microbial peptides reduce the oral microbes, resulting in decreased exposure of periodontal tissues to microbial products. B) The anti-inflammatory pathway: The anti-inflammatory effect of vitamin D arises from the reduction of IL-6 and TNF-α cytokines through the inhibition of NF-kB and the up-regulation of MKP-1. The reduction of these pro-inflammatory cytokines decreases connective tissue destruction by attenuating the stimulation of the matrix metalloproteinases (MMPs). In addition, the reduction of TNF-α and IL-6 down-regulates the RANKL/OPG ratio in stromal osteoblasts. This, in turn, inhibits the differentiation of the osteoclast progenitor cells which results in reduced bone resorption.

Vitamin D plays an important role in calcium homeostasis and is essential for bone growth and preservation.^29^ However, it is well established that its role extends well beyond that, as it has been demonstrated that vitamin D also presents anti-inflammatory effects by modulating both adaptive and innate immunity.^24^ Specifically, 1,25(OH)2D3 has been shown to inhibit antigen-induced T-cell proliferation as well as the differentiation, maturation and function of human monocyte-derived and antigen-presenting dendritic cells.^10^ In addition, it has been found to inhibit the production of several cytokines such as IL-1β, IL-2, IL-6 and TNF-α,^3,33,57,67^ with its inhibitory effects on TNF-α production to be mediated through the reduction of the NFκB kinases activity.^16^ Specifically, in the presence of 1,25(OH)2D3, IκBα phosphorylation is decreased, and the cellular content of this protein is augmented. This prevents NFκB from translocating into the nucleus, leading to the inhibition in the production of TNF-α. Furthermore, the binding of 1,25(OH)2D3 to vitamin D receptors (VDR) has been shown to activate the transcription of vitamin D response element (VDRE). This results in the up-regulation of mitogen-activated protein kinase phosphatase-1 (MKP-1), inactivating the MAP kinases and thus inhibiting the production of TNF-α and IL-6 by macrophages.^64,67^ The vitamin D-mediated reduction of inflammatory mediators may also affect bone resorption. Animal studies have shown that 1,25(OH)2D3 attenuated bone resorption and *P. gingivalis*-induced inflammation through the decrease in expression levels of RANKL and osteoclast related genes as well as the inhibition of pro-inflammatory cytokines including IL-6, IL-12p40 and TNF-α.^42^ However, it should be noted that most of the evidence for the anti-inflammatory effects of vitamin D derives from in vitro studies with clinical human studies being scarce. In a cross-sectional study, serum concentration of 25(OH)D3 was negatively correlated with serum concentration of C-reactive protein (CRP), and in the same study vitamin D supplementation significantly reduced serum concentrations of CRP by 23%.^59^ Also, in a small randomized clinical trial, vitamin D supplementation significantly decreased circulating concentrations of CRP and IL-6 in critically ill patients.^60^ Apart from the anti-inflammatory effects, vitamin D may also present anti-microbial properties. Specifically, VDRs activated by 1,25(OH)2D3 induce the expression of CAMP and β-defensins, which are peptides with anti-microbial activity.^45^ Interestingly, β-defensins are known to exhibit anti-microbial activity against oral microbes including periodontitis-related bacteria such as *Porphyromonas gingivalis, Fusobacterium nucleatum* and *Aggregatibacter actinomycetemcomitans*.^1^ Particularly, Grenier et al^26^ demonstrated that the expression of some genes that encode critical virulence factors of *Porphyromonas gingivalis*, involving adhesins and proteinases, was inhibited by 1,25(OH)2D3. Furthermore, it has been demonstrated that vitamin D induces β-defensin-3 secretion by human gingival epithelium cells (HGE) and human periodontal ligament cells (HPL), reducing the host-cell infectivity by *Porphyromonas gingivalis.*^18^ However, in a clinical study of 855 participants, no association was found between pathogenic oral bacteria including *Porphyromonas gingivalis, Tannerella forsythia, Fusobacterium nucleatum, Prevotella intermedia*, and *Campylobacter rectus* and 25(OH)D concentrations in postmenopausal women.^55^ The authors reported that these findings may be attributable to the species of bacteria assessed, small effect size or a true absence of an association. The potential biological mechanism that connects vitamin D with periodontitis is summarized in Fig 1. Based on the aforementioned anti-inflammatory and anti-microbial effect of vitamin D, many studies have assessed the effects, if any, of vitamin D serum level in the prevalence and severity of periodontal disease and tooth loss.

## Association Between Serum Concentration of Vitamin D, Periodontal Disease Severity and Tooth Loss

Many studies have been conducted to determine the correlation of vitamin D status with periodontitis severity and/or its effect on tooth survival rate. Table 1 presents studies that investigated the correlation between vitamin D and periodontal health.

**Table 1:** Characteristics of the studies reporting on Vitamin D and periodontal health

Study	Population	N of patients	Studied parameters	Findings
Dietrich et al, 2004 CS	Adults aged ≥20 years	11,202	CAL	Inverse association between CAL and serum 25(OH)D for subjects ≥50 years. Men and women in the lowest 25(OH)D quintile had greater CAL (0.39 mm and 0.26 mm respectively) compared to individuals from the highest quintile.
Dietrich et al, 2005 CS	Non-smokers, aged 13 to >90 years	6700	BOP	Inverse association between BOP and 25(OH)D among nonsmokers. Subjects in the highest 25(OH)D quintile had 20% lower odds for BOP compared to subjects in the lowest quintile.
Millen et al, 2013 CS	Postmenopausal women	920	Alveolar crest height (ACH), CAL, gingival bleeding, tooth loss, periodontal disease (CDC/AAP definition)	Women with adequate 25(OH)D3 concentration (>50nmol/l) had 42% lower odds of having ≥ 50% of bleeding sites and 33% lower odds of periodontal disease, compared to those with < 50 nmol/l. No association was found between plasma 25(OH)D and ACH, CAL and tooth loss.
Laky et al, 2017 Case-C	Adults with or without periodontitis	29 cases (periodontitis patients) / 29 controls (healthy)	Periodontitis (defined as ≥ 5 teeth with PD≥5mm), PD, CAL, BOP	Higher percentage of patients with periodontitis were 25(OH)D deficient (<50 nmol/l) compared to healthy subjects. No correlation between serum 25(OH)D levels and CAL, PD and BOP in the periodontitis group.
Abreu et al, 2016 Case-C	Adults with or without periodontitis	19 cases (periodontitis patients) / 19 controls (healthy)	Moderate to severe periodontitis (CDC/AAP definition)	Mean serum 25(OH)D levels were significantly lower in patients with periodontitis (18.5 ± 4.6 ng/ml) than in controls (24.2 ± 7.1 ng/ml) Moreover, for every unit increase in serum 25(OH)D levels, the OR for moderate/severe periodontitis was significantly reduced by 12%.
Boggess et al, 2011 Case-C	Pregnant women with or without periodontitis	117 cases (periodontitis patients) / 118 controls (healthy)	Moderate to severe periodontal disease (defined as ≥15 sites with ≥4 mm of probing depth)	Women with periodontal disease had lower serum 25(OH)D levels and an increased likelihood for vitamin D insufficiency (<75 nmol/l) compared to periodontally healthy women.
Huang et al, 2017 Case-C	Rheumatoid arthritis patients with or without periodontitis	173 cases (periodontitis patients) / 581 controls (healthy)	Periodontitis defined by CAL and BOP	Periodontally healthy patients had a significantly higher means levels of 25(OH)D and the OR for periodontitis was significantly decreased with increased 25(OH)D levels.
Antonoglou et al, 2015b CS	Non-smokers non diabetic adults	1262	Teeth with PD ≥ 4mm, gingival bleeding	No association was found between serum 25(OH)D concentration and number of teeth with PD ≥ 4mm or gingival bleeding. Lower proportion of teeth with PD ≥ 4 mm on patients on the highest vs lowest 25(OH)D quintile and good oral hygiene.
Lee et al, 2015 CS	Korean adults aged >19 y	6011	Periodontal disease (defined as Community Periodontal Index [CPI] ≥3)	Among non-smokers, no association between vitamin D deficiency (25(OH)D ≤ 20 ng/ml) and periodontal disease (CPI ≥ 3), whereas, smokers with vitamin D deficiency were more likely to have periodontal disease. *CPI = 3 (at least one site with PD > 3.5 mm)
Millen et al, 2014 PS	Postmenopausal women with stable 25(OH)D levels (<20nmol/l change between baseline and 5-year follow-up)	442	Periodontal disease progression during 5-year period assessed through changes in alveolar crest height (ACH), CAL, PD and BOP Periodontal disease (CDC/AAP definition), number of teeth	No association between baseline serum 25(OH) levels and periodontal disease progression during 5 year period. At baseline, 25(OH)D concentrations were lower among women with a greater % of BOP, however, no association was found between 25(OH)D levels, periodontal disease severity and number of teeth.
Pavlesen et al 2016 PS	Postmenopausal women	780	Incidence of tooth loss due to periodontal disease during 5 year period	Women with 25(OH)D sufficiency (>50nmol/l) did not have greater tooth loss incidence compared to subjects with 25(OH)D inadequacy/deficiency (<50nmol/l) during 5 year period.
Zhan et al, 2014 PS	Adults aged 20-79 years	1904	Periodontal disease progression defined as CAL ≥ 3mm, tooth loss incidence during 5-year period	Serum 25(OH)D concentration was inversely associated with tooth loss, i.e. a 10 μg/l increase in 25(OH)D decreased the risk for tooth loss by 13%. No association with periodontal disease progression.
Jimenez et al 2014 PS	Adult males aged 40-75 years	42,730	Incidence of tooth loss and periodontitis during 20-year period assessed through questionnaires/ self-report *25(OH)D levels were predicted based on dietary and lifestyle factors and not actually measured	Dose-dependent inverse association between predicted 25(OH)D levels and incidence of tooth loss and periodontitis after adjustment for confounding variables.
Liu et al 2009 Case-C	Adults with AgP, CP and healthy controls	66 AgP/ 52 CP / 60 controls	AgP, CP, bleeding index	25(OH)D levels were higher in AgP compared to controls (29.28 vs 21.60 nmol/l), with no difference between CP and controls. SS association between 25(OH)D and bleeding index in AgP.
Zhang et al, 2013 Case-C	GAgP patients, healthy controls	44 GAgP / 32 controls	GAgP	Higher 25(OH)D levels in GAgP patients compared to controls (25.50 vs 15.25 nmol/l).
Liu et al, 2010 PS	GAgP	19	Plasma and GCF 25(OH)D changes after periodontal treatment	SS decrease in plasma and GCF 25(OH)D levels 2 months after periodontal treatment.
Antonoglou et al 2013 CS	Type 1 Diabetes patients	80	Periodontal disease severity (no/mild vs moderate/severe) Plasma 25(OH)D and 1, 25(OH)2D3 changes after periodontal treatment	Plasma 1,25(OH)2D3 concentration was SS higher in subjects with no or mild periodontitis compared to those with moderate or severe periodontitis. The initial periodontal therapy increased the 1,25(OH)2D3 plasma concentration by 51.9% in patients with moderate or severe periodontitis and by 37.8% in patients with no or mild periodontitis. No association between disease severity and 25(OH)D levels. No changes in 25(OH)D levels after periodontal therapy.
Antonoglou et al 2015a Case-C	Periodontitis patients, healthy controls	55 Periodontitis / 30 controls	Periodontal health status (periodontitis vs healthy)	Patients with low serum 1,25(OH)2D3 were more likely to belong to periodontitis group. 75% of the CP subjects and 30% of the controls were deficient (<50 nmol/l). Serum 25(OH)D was not associated with periodontal health status.

CS: cross sectional; case-C: case-control; PS: prospective; CAL: clinical attachment loss; BOP: bleeding on probing; PD: pocket depth; AgP: aggressive periodontitis; CP: chronic periodontitis; GAgP: generalized aggressive periodontitis; SS: statistically significant; GCF: gingival crevicular fluid.

## Cross-sectional and Case-Control Studies

In a large cross-sectional study of 11,202 subjects (NHANES III data), Dietrich et al^21^ reported an inverse association between clinical attachment loss (CAL) and serum 25(OH)D in men and women ≥ 50 years old. Particularly, men from the lowest quintile of serum 25(OH)D (≤ 40.2 nmol/l) had a mean CAL that was 0.39 mm higher compared to men from the highest quintile (≥ 85.6 nmol/l) after adjusting for multiple covariates including age, smoking and diabetes. In women, the same difference was 0.26 mm. This association was found to be independent of bone mineral density (BMD).^21^ In a subsequent study, the same group of investigators found an inverse association between serum 25(OH)D and bleeding on probing (BOP) among nonsmokers. More specifically, an increase in serum 25(OH)D of 30 nmol/l was associated with 10% lower odds for BOP.^22^ In both studies, the authors reported that the findings may be explained by the anti-inflammatory effects of vitamin D. In another cross-sectional analysis of 920 postmenopausal women (Buffalo OsteoPerio Study), an association was reported between plasma 25(OH)D and periodontal disease.^50^ Women with adequate 25(OH)D levels (≥ 50 nmol/l) had 33% lower odds of having periodontal disease (CDC/AAP definition) and 42% lower odds of having ≥ 50% of gingival bleeding sites, when compared to women with inadequate 25(OH)D levels. Nevertheless, there was no association between 25(OH)D levels, CAL and alveolar crest height (ACH), with the authors suggesting that vitamin D levels may influence inflammatory markers, but no markers of chronic periodontal disease. In a case control study of 58 patients,^39^ a higher percentage of patients with periodontitis (defined as minimum of 5 teeth with PD ≥ 5 mm) had deficient 25(OH)D levels (<50 nmol/l) compared to non-periodontitis patients (48.3% vs 13.8%, respectively). However, no significant correlation was found between 25(OH)D levels and PD, CAL and BOP. A similar study by Abreu et al^2^ in Puerto Rican adults also reported mean serum 25(OH)D levels to be significantly higher in the healthy patients compared to periodontitis patients. In another case control study of 325 pregnant women, vitamin D insufficiency defined as <75 nmol/l, was independently associated with moderate to severe periodontal disease (defined as ≥ 15 sites with ≥ 4 mm of probing depth). It was suggested that insufficient maternal vitamin D levels might constitute a risk factor for periodontal disease during pregnancy, with vitamin D supplementation, representing a potential therapeutic strategy.^11^ Finally, Huang et al,^30^ in a retrospective analysis of 754 patients with rheumatoid arthritis, also reported that the odds of periodontitis among subjects was significantly decreased with higher 25(OH)D levels. Contrary to the aforementioned studies, in a cross- sectional study of a non-smoking population of 1262 individuals (30 to 49 years old), Antonoglou et al^7^ did not find any association between serum 25(OH)D levels and number of teeth with periodontal pockets (PD ≥ 4 mm) or gingival bleeding.^7^ Similarly, Lee et al,^40^ in a sample of 6011 subjects, did not report any association between vitamin D deficiency (25[OH]D ≤ 20 ng/ml) and periodontal disease (defined as community periodontal index [CPI] ≥3) among nonsmokers. In current smokers, however, subjects with vitamin D deficiency were more likely to have periodontal disease (CPI ≥ 3). The authors concluded that smoking could modify the effect of vitamin D on periodontitis.

## Prospective Studies

In a 5-year prospective study^49^ of 442 postmenopausal women with stable 25(OH)D levels (< 20 nmol/l change between baseline and follow-up), no association was reported between baseline 25(OH)D levels and periodontal disease progression assessed through changes in ACH, CAL, PD and BOP, after adjustment for confounding variables. Authors claimed that vitamin D supplementation for prevention of periodontal disease progression is not justified based on the results of this study. Pavlesen et al^54^ in another study investigating the association of 25(OH)D concentration and a 5-year incidence of tooth loss due to periodontal disease, reported no difference between adequate (≥ 50 nmol/l) and inadequate/deficient (<50 nmol/l) 25(OH)D levels and tooth loss incidence. However, another prospective study of 1904 participants and a 5-year follow-up found an inverse association between serum 25(OH)D concentration and tooth loss.^65^ Each 10-µg/l increase in 25(OH)D levels, was associated with a 13% lower risk of tooth loss after multivariate adjustment, with the observed association to be partially explained by changes in the periodontal status. However, no association was observed between serum 25(OH)D and periodontal disease progression expressed as CAL ≥ 3 mm. The authors concluded that vitamin D might have a protective role on tooth loss, with the effect partially mediated by its effect on periodontitis. Finally, in another prospective study of 42,730 men, predicted 25(OH)D levels (based on dietary and lifestyle factors) were associated with incidence of tooth loss and periodontitis over 20 years of follow-up.^32^ Men with a predicted 25(OH)D score in the highest quintile exhibited a significantly lower risk of tooth loss and periodontitis compared with men in the lowest quintile after adjustment for covariates. Each 10 nmol/l increase in the predicted 25(OH)D score was also associated with a 10% significantly lower risk of tooth loss.

## Aggressive Periodontitis

Different outcomes have been reported on the association between 25(OH)D levels and aggressive periodontitis. In a cross-sectional study, Liu et al^44^ reported higher plasma 25(OH)D levels for aggressive-periodontitis patients compared to healthy individuals (29.8 vs 21.60 nmol/l), whereas no difference was noted between chronic periodontitis patients and healthy controls. In another cohort of 44 GAgP patients, Zhang et al^66^ also reported higher plasma 25(OH)D levels when compared to healthy individuals (25.50 vs 15.25 nmol/l). The same group of investigators in a subsequent study of 19 patients with GagP reported that plasma and gingival crevicular fluid levels of 25(OH)D significantly decreased 2 months after initial periodontal therapy.^43^ The authors hypothesized that 25(OH)D might be generated by inflamed periodontal tissues based on the study findings, including 1. local 25(OH)D levels were considerably higher compared to systemic levels, 2. positive correlation between local and systemic 25 (OH) levels, and 3. decrease in 25(OH)D levels after initial periodontal therapy.

## Association of 1,25(OH)2D3 Levels and Periodontitis

Antonoglou et al^5^ reported a positive association between serum 1,25(OH)2D3 and periodontal health in type 1 diabetes patients after adjusting for covariates including smoking, plaque and HbA1c among others. Subjects with high 1,25(OH)2D3 levels were more likely to belong to the group of subjects with no or mild periodontitis than to the group of moderate or severe periodontitis. Such an association was not found between 25(OH)D levels and periodontal disease severity as well as between 1,25(OH)2D3 levels and disease severity when only nonsmokers were included. After anti-infective mechanical periodontal therapy, an increase was noted in mean serum 1,25(OH)2D3 levels for all individuals irrespective of disease severity. The same pattern was not followed however for 25(OH)D levels.^5^ In another case-control study by the same group of investigators,^6^ subjects with low 1,25(OH)2D3 were more likely to belong to the periodontitis group than the periodontally healthy group. Similar to the previous study however, no association was noted between 25(OH)D levels and periodontal health.

## Effects of Vitamin D and Calcium Oral Supplementation on Periodontal Disease

Krall et al^37^ examined the incidence of tooth loss during a 3-year period in patients under calcium and vitamin D supplementation, as well as during a 2-year follow-up period after discontinuation of supplements. They found that during the 3-year period, 27% of the subjects in the placebo/non-supplemented group and 13% in the supplemented group lost one or more teeth. During the follow-up, 59% of the subjects who were at the lower calcium intake level (less than 1000 mg per day) lost one or more teeth compared with 40% of the subjects who were at the higher calcium intake level. Hence, they concluded that calcium and vitamin D supplements were associated with a lower risk of tooth loss in elderly men and women. However, due to the design of the study, it was not possible to separate the effects of calcium and vitamin D. In another cross-sectional study, Miley et al^48^ studied whether vitamin D and calcium supplementation (≥ 400 IU/day and ≥ 1000 mg/day respectively) for more than 18 months in subjects receiving periodontal maintenance therapy could affect their periodontal status. It was reported that all periodontal parameters (PD, CAL, BOP, gingival index, furcation involvement) were better in individuals who took oral supplementation compared to those who did not, with the differences being borderline significant when all parameters were considered collectively (p = 0.08). The authors concluded that vitamin D and calcium supplementation could be advocated as a component in the management of periodontal disease, as there is a trend toward better periodontal health. In a subsequent study, the same group of investigators examined whether these differences persisted over a 1-year period in the same cohort of subjects, enrolled on maintenance programs. All parameters for both groups (with and without calcium and vitamin D supplementation) improved throughout the study period but remained worse among those who did not take the supplements, but the differences were not statistically significant.^23^ The authors concluded that vitamin D supplementation may have a modest positive effect on periodontal health, stressing the need to assess the effects of higher vitamin D supplementation dosages on the periodontium. In a subsequent study, Alshouibi et al^4^ assessed the effects of total vitamin D intake (from food, supplements and multivitamins) on periodontal health of 562 adult males. It was reported that the odds of severe periodontitis among men consuming ≥800 IU/day were 0.67 relative to those consuming <400 IU/day. An inverse association between vitamin D intake and alveolar bone loss was also noted, with each 100 IU increment in daily total vitamin D intake to be independently associated with reduced odds for moderate to severe ABL. The authors concluded that vitamin D intake may have a protective effect on periodontal disease and supported adherence to the daily recommendation of vitamin D intake for older adult of at least 800IU/day due to its impact on periodontal health.^4^ Another study assessed the effects of vitamin D status and vitamin D supplementation on periodontal surgery.^9^ Vitamin D sufficiency (serum levels ≥20 ng/ml) at the time of surgery resulted in an average of 1.35 mm greater CAL gain and 1.4 mm greater PD reduction compared with deficient patients at 12 months. Calcium and vitamin D supplementation (1000 mg and 800 IU daily respectively) initiated at 3 days prior to surgery and continued for 6 weeks after, failed to prevent the negative clinical outcomes associated with baseline deficiency. Positive effects of vitamin D supplementation on gingival inflammation have also been reported in a recent randomized controlled trial.^28^ Participants were stratified in four groups and each group received a different dose of daily vitamin D supplements (2000IU, 1000IU, 500IU and placebo drug). Re-evaluations after 1, 2 and 3 months indicated that subjects receiving the highest dose of vitamin D had the greatest improvement in gingival scores, with the authors attributing these outcomes to the anti-inflammatory effects of vitamin D.

## Vitamin D Receptor Polymorphisms and Their Association with Periodontal Disease

As periodontitis is considered a multifactorial disease with environmental and genetic risk factors,^36,46^ genetic studies have examined the vitamin D receptor (VDR) gene located in chromosome 12 as a potential candidate gene associated with periodontal disease due to its effects on bone metabolism^38^ and immune system.^61^ A number of studies^8,13,17,19, 27,31,41,47,53,56,58,62,14^ have investigated VDR polymorphisms at restriction fragment length polymorphisms positions Taq-I, Bsm-I, Apa-I and Fok-I and their association with aggressive (AgP) and/or chronic periodontitis (CP) in different ethnic populations (Table 2). As it can be observed, there is inconsistency regarding the association of the various VDR gene polymorphisms and periodontal disease. For example, five studies reported that carriage of the (VDR Taq I) (T) allele was associated with CP in various ethnic populations,^8,13,47,56,62^ with some suggesting that the t-allele may be protective against periodontal disease^13^ whereas, three other studies reported that carriage of the t-allele was associated with increased susceptibility to CP when compared to T-allele carriage.^17,58,31^ In a longitudinal study, Inagaki et al^31^ demonstrated an association between the VDR Apa I AA polymorphism and periodontal disease severity and progression, whereas three other studies found no association between the Apa I and periodontitis.^41,58,62^ Furthermore, Park et al^53^ and Li et al^41^ detected a significantly higher frequency of FokI FF genotype in the generalized AgP patients, in Korean and Chinese subjects respectively, whereas three other studies^56,58,62^ did not find any association between Fok I polymorphisms and periodontal disease. In a meta-analysis of 15 studies in VDR gene polymorphisms and periodontal disease, Deng et al^20^ reported that CP cases had a higher frequency of AA (Apa-I) and TT (Taq-I) genotype and lower frequency of bb (Bsm-I) genotype in Asians, whereas no association was found in Caucasians or in AgP cases. In a subsequent meta-analysis, Chen et al^15^ found that the t-allele (Taq-I) may be protective for CP but not for AgP in Asians, whereas the allele F (Foq-I) appeared to be a risk factor for AgP rather than CP in Asians. In contrary to the previous study, no association was found for Bsm-I and Apa-I polymorphisms and periodontitis.^15^ Possible reasons for these differences may include variations in study design, the small sample size of some studies, the heterogeneous populations, the variation in environmental factors between geographically separated areas and different linkage disequilibrium and haplotype blocks in populations.^15^

**Table 2:** Characteristics of the studies reporting on vitamin D receptor polymorphisms and periodontal disease

Hennig et al 1999 CS	Caucasian	69/72	L-AgP	Taq I	Carriage of the less frequent t-allele increased the risk for developing L-AgP.
Tachi et al 2003 Case-C	Japanese	74/94	CP	Taq I Fok I	The Taq I TT genotype and the T-allele were found to be associated with CP, independently of smoking and diabetes. Fok I was not found to be associated with CP.
Inagaki et al 2003 Longitudinal	Not mentioned	125/-	Disease progression	Apa I Taq I	ABL, CAL and tooth loss occurred at higher rates in the AA genotype compared to the Aa and aa genotypes. Presence of t allele was associated with more severe CAL in the single gene analysis. BOP did not vary significantly by VDR genotype. Combination of Apa I and Taq I genotypes modulated the progression of CAL and ABL.
De Brito Junior et al 2004 Case-C	Brazilian	69/44	CP	Taq I Bsm I	Carriage of the t-allele (Tt or tt) increased susceptibility to CP. No significant differences were found in the distribution of the Bsm I between cases and controls.
Brett et al 2005 Case-C	Caucasian	57 (CP) / 51 (AgP) / 100	CP, AgP	Taq I	The Tt and tt genotypes were more prevalent in controls compared with patients with chronic disease suggesting that the t-allele may be protective against periodontal disease.
Park et al 2006 Case-C		93/143	AgP	FokI Taq I Bsm I	The Fok I FF genotype was detected with a significantly higher frequency in the G-AgP patients. TaqI and Bsm I polymorphisms were not found to be associated with AgP.
De Souza et al 2007 Case-C	Caucasian, Afro-American, Mulatto	113/109	CP	Taq I Bsm I	No differences in the allele or genotype distribution of Taq I or Bsm I polymorphisms were observed between the groups. Allele T for Taq I polymorphism was associated with increased gingival index.
Li et al 2008 Case-C	Chinese	51/53	AgP	Bsm I Taq I Apa I Fok I	The frequency of Fok I FF genotype was significantly higher in GAgP patients than in healthy control subjects. The frequency of allele F was also higher in the GAgP group. No evidence that VDR Bsm I, Taq I or Apa I polymorphisms were associated with GAgP.
Wang et al 2009 Case-C	Chinese	107/121	CP	Bsm I Taq I Apa I Fok I	The Taq I TT genotype was more prevalent in patients with severe CP compared to controls. The T-allele was more frequent in patients with CP than in controls. For the Bsm I, Apa I and Fok I polymorphisms, neither genotype, nor allele was associated with severe CP.
Martelli et al 2011 Case-C	Italian	115(CP)/58 (AgP) /65	CP, AgP	Taq I	Patients with Taq I TT genotype were more susceptible to CP compared to patients with tt genotype, and more susceptible to AgP compared to patients with Tt genotype. T-allele was correlated with CP whereas t allele was correlated with AgP. The authors proposed that VDR Taq I polymorphisms could be a helpful differential test for the discrimination of CP and AgP.
Baldini et al 2013 Case-C	Caucasian	42/39	CP	Taq I	The T-allele of VDR Taq I polymorphisms showed a high prevalence in subjects with CP. TT and Tt genotypes were correlated with the development of CP, whereas tt genotype was more frequent in healthy subjects.
Tanaka et al, 2013 Case-C	Japanese	131/1019	CP	Bsm I Taq I Apa I Fok I	The tt genotype of Taq I polymorphisms was associated with increased susceptibility to CP, compared to Tt and TT genotypes. Apa I, Fok I and Bsm I were not significantly associated with periodontal disease.
Chantarangsu et al, 2016 CS	Thai	370 no/mild, 725 moderate, 365 severe	CP	Bsm I Taq I Apa I Fok I	Fok I polymorphism was associated with severe CP (OR = 1.9). A synergistic interaction was revealed between Fok I polymorphism and smoking, since genotype positive smokers were even more likely to have severe CP.

CS: cross sectional; Case-C: case-control; CAL: clinical attachment loss; BOP: bleeding on probing; PD: pocket depth; AgP: aggressive periodontitis; CP: Chronic periodontitis; GAgP: aggressive periodontitis; LAgP: localized aggressive periodontitis; SS: statistically significant; GCF: gingival crevicular fluid; ABL: alveolar bone loss.

## Discussion

There is conflicting evidence regarding the association of vitamin D and periodontal disease. The majority of cross- sectional studies identified reported that higher serum concentrations of 25(OH)D were associated with lower disease prevalence,^2,11,30,39,50^ lower mean CAL^21^ and BOP.^22,50^ However, some of the same studies and others, when they reported specifically on markers of periodontal disease severity and inflammation, did not find any association between 25(OH)D levels and PD,^7,39^ CAL,^39,50^ BOP,^7,39^ and alveolar bone loss.^50^ This may be attributed to the design of the aforementioned studies and the presence of a minor correlation which cannot be highlighted through the methods applied. Conflicting results were also reported for longitudinal studies, with some studies reporting no association between serum 25(OH)D concentration, periodontal disease progression^49,65^ and incidence of tooth loss,^54^ whereas others reported an inverse association with periodontitis^32^ and tooth loss incidence.^32,65^ The majority of the studies that found a positive association of vitamin D with periodontium attributed it to the potential anti-inflammatory effects of vitamin D.^21,22,50^ Nevertheless, one should not ignore studies on AgP that associated 25(OH)D levels with increased inflammatory response. More specifically, three studies were identified, all from the same group of investigators.^43,44,66^ Higher 25(OH)D levels were reported for AgP patients compared to healthy individuals,^44,66^ which comes in contrast with outcomes reported for 25(OH)D and CP. In an effort to explain such outcomes, authors suggested that 25(OH)D might be generated by inflamed periodontal tissues in AgP patients.^43^ It is possible however, that in these studies, the small sample size and the Chinese ethnicity of the participants may have contributed to the observed conflicting results. Also, the anti-inflammatory effects of vitamin D may not be as evident in AgP patients. Whether 25(OH)D may have different biological functions in CP and AgP patients needs to be further investigated.

Even if 1,25 (OH)2 D3 is a biologically more active molecule than 25(OH)D, only two studies were identified reporting on the effects of 1,25(OH)2D3 on periodontal health.^5,6^ More specifically, patients with higher 1,25 (OH)2 D3 were more likely to be periodontally healthy, however, on these studies, such pattern was not seen for 25(OH)D levels. Such discrepancy between 1,25(OH)D3 and 25(OH)D effects on periodontal health may warrant a shift on the studies reporting on vitamin D and periodontitis towards investigating more 1,25(OH)D3 as a potential factor affecting disease severity and progression.

In the majority of the studies identified, vitamin D supplementation had a positive effect on periodontal health. More specifically, vitamin D supplementation was reported to enhance tooth retention and improve radiographic and clinical periodontal parameters such as gingival inflammation and CAL.^4,28,37,48^ However, in some studies, vitamin D supplementation was accompanied also by an increased intake of calcium.^23,37,48^ Since the beneficial effects of the two supplements on the periodontal status could not be separated, some of these results should be interpreted with caution. Also, as various levels of vitamin D supplementation were assessed in the studies identified, ranging from <400IU/day^4^ to 2000IU/day,^28^ optimal vitamin D intake levels to maximize its positive effects on the periodontium need to be further investigated. Finally, no definite conclusion on the effects of VDR polymorphisms and periodontal disease can be drawn, as shown by the conflicting outcomes of the studies identified. Variations in the study design and the small sample size of some studies may have contributed to this inconsistency. However it is also possible that some polymorphisms may have a different impact on periodontal disease status depending upon the ethnicity of the population and the severity of the disease.

## Conclusions

Vitamin D, apart from regulating calcium homeostasis, may modify the immune system and exert some anti-inflammatory and anti-microbial effects which could influence the periodontal disease severity and progression. However, available evidence so far is conflicting regarding the effects of 25(OH)D on periodontal disease with cross-sectional and longitudinal studies reporting either beneficial effects of vitamin D on periodontal health or no effects whatsoever. There is also limited evidence that supports a positive association of periodontal health with 1,25(OH)2D3 and vitamin D supplementation with a small number of studies reporting that vitamin D supplementation could enhance tooth retention and periodontal health. Larger, multisite longitudinal studies are warranted to further investigate the association of vitamin D to periodontal health and the benefits of vitamin D oral supplements, especially given their low cost and ease of access.
